# Crop Production and Security in Ningjin County of the North China Plain

**DOI:** 10.3390/foods12112196

**Published:** 2023-05-30

**Authors:** Shuang Wang, Lin Zhen, Yunfeng Hu

**Affiliations:** 1 Institute of Geographical Sciences and Natural Resources Research, Chinese Academy of Sciences, Beijing 100101, China; wangs.17s@igsnrr.ac.cn (S.W.); huyf@lreis.ac.cn (Y.H.); 2 University of Chinese Academy of Sciences, Beijing 101408, China; 3 State Key Laboratory of Resources and Environmental Information System, Beijing 100101, China; 4 Key Laboratory of Carrying Capacity Assessment for Resource and Environment, Ministry of Natural Resources, Beijing 100101, China

**Keywords:** crop planting structure, crop production and security, influencing factors, farming practices

## Abstract

Stable growth in grain production is a critical challenge to ensure food security in North China Plain (NCP), an area dominated by smallholder farming. Food production and security of NCP largely depend on how smallholders farm their land. This study took Ningjin County of the NCP as an example to describe the characteristics of crop planting structure and the changes in crop production based on household surveys, statistics, various documents, and literature by descriptive statistics, calculation of crop self-sufficiency, and curve fitting, and aimed to reveal crop security and the influencing factors of crop production at the household level. The results were as follows: (1) Wheat and maize sown area accounted for 61.69% and 47.96% of the total sown area of crops during 2000–2020, increasing at a rate of 3.42% and 5.93%, respectively. Their planted areas increased from 27.52% and 15.54% in 2000 to 47.82% and 44.75% in 2020, respectively. (2) The self-sufficiency rate of maize showed a significant upward trend and reached its peak in 2019. the self-sufficiency rate of wheat also showed an increasing trend, from 192.87% to 617.37%, which indicates that wheat and maize can meet food self-sufficiency and the per capita grain yield is in a safe state. (3) The trends on wheat yield and fertilizer initially grew, then decreased, closely resembling an inverted “U”, while the maize yield showed a pattern of increasing first and then basically remaining stable, similar to an “S” shape. A turning point for fertilizer use (550 kg/ha) was identified, indicating the limits of fertilizer use to increase yield. The national agricultural production and environmental protection policies, continuous improvement of crop varieties, as well as the farmers’ traditional practices have significant impacts on crop production. This study will enhance management practices for improved yield, which can support the integrated management of agricultural production in intensive agricultural areas.

## 1. Introduction

As the second among the 17 Sustainable Development Goals (SDGs), the importance of food security has been highlighted by the United Nations [1], which should be guaranteed by comprehensive management practices that synchronously emphasize on production and environmental issues [2]. Globally, 2.37 billion people lacked access to adequate food in 2020 as a result of the COVID-19 pandemic (approximately 40% of whom were severely food insecure), an increase of 320 million from 2019 [3]. Undoubtedly, the COVID-19 pandemic has further exacerbated world hunger [4,5], calling for enhanced food production. China contributes to global food security by feeding roughly 20% of the world’s population on less than 9% of arable land [6]. Since 2004, China’s grain production achieved increases for 16 consecutive years, and China’s total grain production reached 669.49 million tons in 2020, accounting for 24.17% of the global grain production [7]; among this, the outputs of wheat and maize were 13,425 and 260.67 million tons [8], accounting for 23.09% and 17.30% of global production, respectively, meaning that China ranks first and second among the major wheat- and maize-producing countries [7]. This contributes to the solution of global food security issues. The North China Plain (NCP), as one of the most densely populated regions in China, is the largest alluvial plain with excellent conditions for agriculture [9,10]. The NCP is a major food-producing region in China, in which the production of wheat and maize accounts for 75% and 35% of China’s total wheat and maize output, respectively, which is crucial to China’s food security [11]. However, the COVID-19 pandemic has further exacerbated world hunger [4,5]; therefore, stable growth in grain production is a critical challenge to ensure food security in the NCP, an area dominated by smallholder farming [2,10,12].

Natural conditions (such as water, temperature, sunshine, and soil) [13,14], planting structure [15], crop varieties [16], agricultural policies issued by the national and local government [17], and farming practices (such as fertilizer and irrigation) [18,19] have shown considerable impact on crop yield. China’s food production has benefited from farming practices, increasingly effective agricultural technologies and continuous improvement of crop varieties, expanded irrigation infrastructure, and massive increases in the usage of synthetic fertilizer [10,20]. Fertilization, irrigation, and climate have a large influence on grain yields [21], and most grain yields can increase 45–70% by fertilization and irrigation [22]. Since the agricultural policy was enacted, such as “Household Responsibility System”, which distributes land into individual households, smallholder farmers are the major driving force for food production increases since the 1980s [23]. An intensive production practice with high input had been adopted by smallholders in NCP since the 1980s, the usage of fertilizer had reached 550–600 kg N ha^−1^ y^−1^ [24] and the amount of irrigation reached 400–700 mm y^−1^ [25], while the grain output only increased 2.8-fold when the application of fertilizer increased by 5.1-fold [26], meaning that smallholders operate high-input and low-efficiency practices in NCP [2]. However, these practices, such as irrigation and fertilizer application, not only contribute to crop yield but also result in a series of environmental issues [27,28,29].

Currently, four aspects are the key fields of attention for research on crop production in the NCP. The first is a long-term series of grain yield and crop planting structure studies, and as science and technology have advanced, 3S technology has gradually become the main means of monitoring the changes in planting area and structure [26,30]. Second, research on the water requirements for crop production at different stages [31], irrigation water and its sources [32], and the impact of irrigation on increased crop yield has been extensively conducted [22]. Third, research has been carried out on the application status and influence of different chemical fertilizers. The findings have indicated that nitrogen (N) fertilizer has contributed to increasing grain production [33], and various experiments have demonstrated that decreasing the N application rates and increasing N use efficiency during maize/wheat cultivation may be achieved without significantly affecting crop yield [33,34,35]. Lastly, studies on changes in the planting environment during food production have found that surface water and groundwater vary at rates of −0.6 and −0.5 cm/y, respectively [36], that groundwater quality is deteriorating [34], and that soil salinization is increasing [37]. Furthermore, environmental problems arising from agricultural production also have aroused tremendous concern, for example, water deficit [10], N and P losses [27], ammonia volatilization [28], soil acidification [29], nitrate leaching [38], groundwater contamination [39], soil pollution [40]. Therefore, how to achieve the sustainable development goal that grain production should both improve grain output and conserve the environment is essentially vital, especially in intensive agricultural regions [41,42].

Additionally, previous studies have been dominated by the analysis of agricultural production input-output at the macro scale and the way to enhance smallholder productivity [43,44], which finds that the emergency yield ratio and emergency sustainability index for maize monocropping are 13.7% and 21.1% greater, respectively, than those for intercropping systems. In fact, in intensive farming areas where smallholder farmers are the primary producers, how and how much farmers utilize irrigation and fertilization directly affects crop yield and the agricultural environment. Thus, affecting the sustainability of agricultural production that cannot be accomplished without the large communities of small-scale farmers [2]. Therefore, research on food security at the household level is relatively extensive and important [44].

To narrow the research gaps, this research aimed to (1) reveal the characteristics of crop planting structure in Ningjin County, (2) describe the changes in crop production and security, (3) explore the influencing factors of crop production, which can support comprehensive management for food production, especially for intensive agricultural regions, and also offer a scientific justification for the sustainable development goal that grain production should both improve grain output and conserve the environment.

## 2. Materials and Methods

### 2.1. Study Area

**Figure 1:**
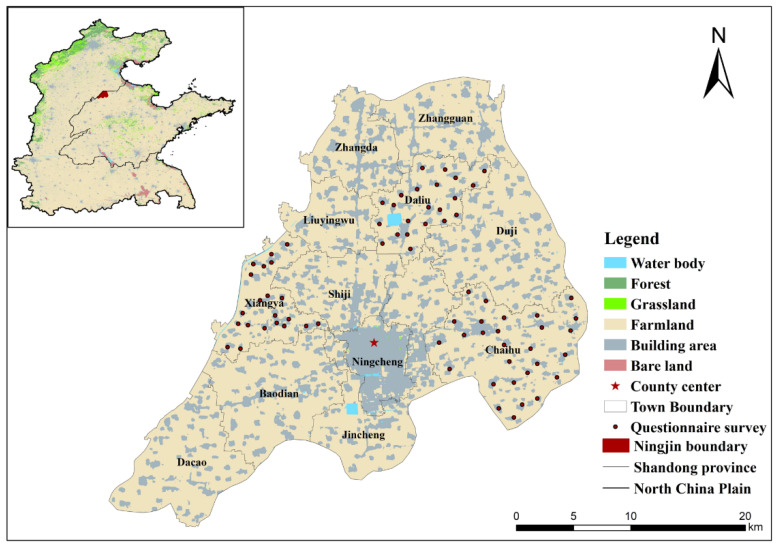
Land cover of Ningjin County in 2020. Note: The land cover data come from the Resource and Environment Data Cloud Platform, Chinese Academy of Sciences (http://www.resdc.cn, accessed on 1 January 2021).

Ningjin County, which has an area of 833 km^2^, is situated at 37°31’–37°51’ N 1163°0’–117°02’ E in the Yellow River flood plain in the eastern portion of the North China Plain (Figure 1). The county has 9 towns with 856 villages throughout 2000–2020 and the average annual population was 471,014, among which the agricultural population was 393,502, accounting for 83.54% of the total population. Thus, the average population density is 555/km^2^. Browning and tidal soil made up the majority (99.7%) of the county’s available soil and are ideal for growing crops. In 2020, the area of farmland, forest, grassland, water body, and built-up areas was 62,477.84, 72.15, 11.24, 376.88, and 20,391.91 ha, accounting for 74.98%, 0.09%, 0.01%, 0.45%, and 24.47% of total area, respectively. The spatial pattern is mainly dominated by farmland with a scattered distribution of other land use types (Figure 1), making it the nation’s primary center for food production.

**Figure 2:**
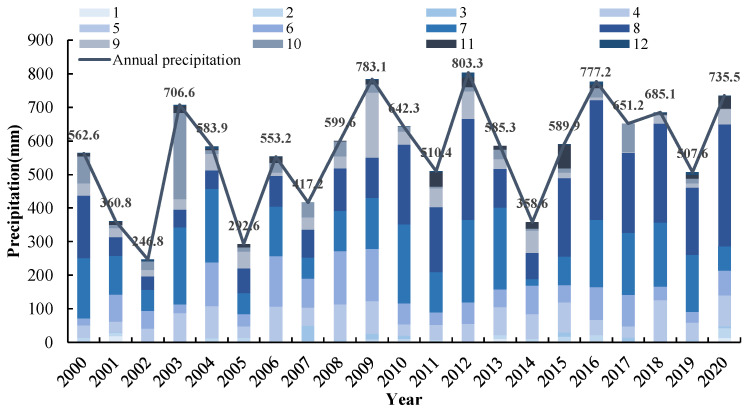
Annual precipitation and its distribution among monthly of Ningjin County throughout 2000–2020. Note: The precipitation data come from two weather stations of a Chinese meteorological data-sharing service (http://cdc.cma.gov.cn/, accessed on 1 May 2021).

The annual average temperature was 12.5 °C throughout 2000–2020, with July as the hottest month (32.3 °C) and January as the coldest month (−2.75 °C). On average, 569.18 mm of precipitation falls each year, among which July and August roughly account for 55.29% of the total (Figure 2). Only 12.70% of that falls during the most vital water demand season for crops in this region, which is from March to May. The annual evaporation is 2126.8 mm, and the evaporation from April to June accounts for 45% of the annual evaporation. The uneven distribution of precipitation coupled with high evaporation leads to a generally dry climate in the area, with spring in particular having extremely dry weather.

This study was carried out in Ningjin County of Dezhou City, Shandong Province, on the NCP, for the following reasons.

**Figure 3:**
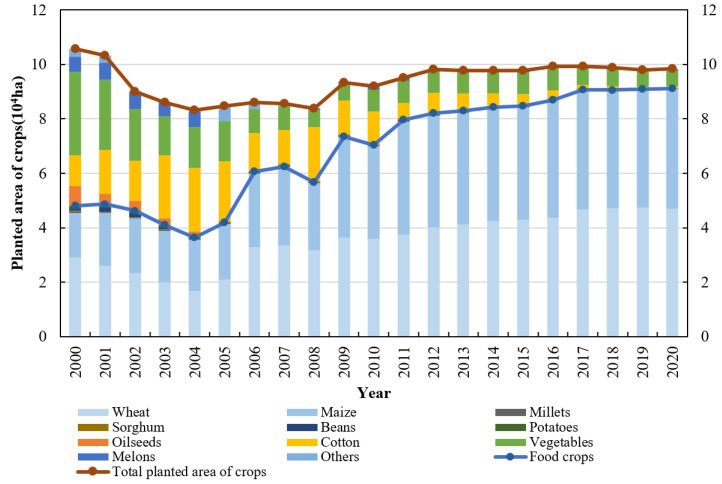
Crop planting structure in Ningjin County throughout 2000–2020.

(1) Ningjin County is the primary agricultural region and a hub for grain production [41]. It is located in the center of the NCP, with farmland accounting for 74.98% of the total area. In total, 25,300 ha of high-standard farmland had been established by 2021, accounting for 45.12% of the county’s arable land and 51.08% of the basic farmland [45]. In 2019, the grain yield per unit of area was 6925.65 kg/ha, much higher than the national grain yield of 5715 kg/ha [46].
(2) Crop production is the primary economic activity. Cereal crops such as winter wheat and summer maize are the principal crops, which occupy 92.58% of the arable land, followed by vegetables (5.91%) and other crops, such as cotton, chives, cucumber, eggplant, and chili (1.51%) (Figure 3). All crops are irrigated with groundwater and the water from Yellow River, at proportions of 41.83% and 58.17%, respectively [45].

(3) The farmers in the area are predominantly small-scale subsistence farmers [47,48]. The average annual population from 2000 to 2020 was 469,700, among which the agricultural population was 396,700, accounting for 84.46% of the total population [40]. In 2020, the per capita cultivated land in Ningjin County was 0.13 ha, which is higher than the national level (0.09 ha) and that of Shandong Province (0.06 ha) [49]. The main production mode is the family as the production unit.
(4) The main economic activity is agricultural production. The per capita disposable income of rural residents is 13,969.17 RMB [45]. In addition to agricultural income, the non-farm income mainly depends on off-farm work to improve economic level.

Therefore, the grain production mode of Ningjin County is representative of the NCP, meaning it can reflect the grain production status of the main grain-producing regions. Thus, exploring the mode and influencing factors of grain production in Ningjin County is the basic guarantee of ensuring the stable production of grain and maintaining food security [47,48].

### 2.2. Data Collection and Methods

#### 2.2.1. Data Collection

The data sources in this study mainly include primary and secondary data.

(1) Collection of secondary data

The secondary data mainly include the statistical yearbook of Ningjin County [45], documents provided by the Agricultural Bureau of Ningjin County [45], the statistical yearbook of Shandong Province [49], and literature, which are primarily used to analyze the crop planting structure, crop production and security, and influencing factors. Specifically, the following data were included: planted area of various crops, per unit yield of wheat and maize, resident population, total yields of wheat and maize, and per capita wheat and maize consumption.

(2) Collection of primary data

The primary data came from a household survey of farmers, conducted in 2021 by online survey and a telephone return visit, and in 2011 and 2001 by a previous practice of the research group [40,47,48]. The household survey was used to explore the farmers’ practices’ (including fertilizer and irrigation) impact on crop production.

A household survey, designed to assess farmers’ existing cropping patterns and crop production practices, such as fertilizer use and irrigation, was one of the major sources of information and was collected using a standard questionnaire. Supplementary Questionnaire includes the following information: the personal information of the respondent (Item 1), crop information for analysis types, cultivated area and unit yield (Item 2), irrigation information for analysis source, frequency and amount (Items 3–6), farmer’s cognition for analysis willingness of cultivation (Items 7–11, 26–28), fertilization information for analysis type, frequency, and amount (Items 12–18), and pesticides information for analysis type, frequency, and amount (Items 19–25).

The survey area in 2021 was the same as the research area in 2011 and 2001, including Chaihudian, Daliu, and Xiangya communes that can accurately represent the general situation of the county in terms of geographic and socio-economic conditions as well as farming practices [40].

In order to be consistent with the data of 2011 and 2001 by the research team during field research, the survey scope and the number of questionnaires in 2021 were set with reference to 2011 and 2001 [40]. After deciding on the number and area of the survey, we made contact with nearby farmers with university degrees and chose one of them to serve as our key contact person. Prior to the questionnaire survey, we performed three training sessions via online video and phone for the contact person and his family members (six individuals in total), during which we thoroughly described the questionnaire’s contents and how to issue and fill it out. For the pre-survey, the contact person was asked to distribute 10 questionnaires on their own. In accordance with the mobile number on the pre-survey questionnaire we collected, we called the respondent again and confirmed the information on the questionnaire. The pre-survey questionnaire had a 100% effective rate.

Contact persons and their families, therefore, were requested to complete a formal questionnaire survey on this basis. According to the research of Zhen et al. (2005) [47], a simple random sampling method was adopted to select households in a questionnaire survey, which can avoid subjective influence and reflect the situation of the study area more objectively [40,47,48]. A total of 240 farmers were chosen for the survey, which included 66, 56, 75, and 43 houses in each of the four villages of Dongliu, Daliu, Dageng, and Daxin. After gathering the questionnaires, we called each person individually. The final effective questionnaires were 220, with an effective rate of 91.67%.

In order to facilitate an analysis and comparison of the usage of various fertilizers in 2001, 2011, and 2021, the data were converted into the contents of N, P_2_O_5_ and K_2_O. The conversion coefficients are shown in Table 1.

#### 2.2.2. Data Analysis Methods

In this paper, planted areas of various crops were analyzed based on descriptive statistics in order to reveal the structure of crop planting, and from the perspective of per unit yield and per capita yield to analyze major crop production also based on descriptive statistics. Moreover, crop self-sufficiency was calculated to quantitatively measure food security based on the data on crop production and consumption. In order to explore the quantitative impacts between fertilizer use and crop yield, a curve simulation of crop yield and fertilizer use throughout 2000–2020 was carried out based on the Python programming language [50,51], the detailed calculation of which is as follows:

(1) Calculation of crop self-sufficiency

The crop self-sufficiency rate is an important indicator for measuring food security, especially intensive grain-producing areas. It is based on the relationship between crop production and consumption and reflects whether the crop is balanced in a typical region.

(1) $${CSS}_{in}=\frac{{TCP}_{in}}{{TCC}_{in}}\times 100\%$$

where CSS represents the crop self-sufficiency, i represents type i crop (including wheat and maize in this paper), n represents a certain year (containing 2000–2020 in this paper), TCP represents total yields of wheat and maize, and TCC represents total consumption of wheat and maize.

(2) $$T{CC}_{in}={CC}_{in}\times {RP}_{n}$$

where CC represents wheat and maize consumption per capita and PRP represents population (the resident population is used in this paper). Due to data limitations, we used the per capita crop consumption of Shandong Province instead of Ningjin County.

(2) Curve fitting

Firstly, we drew a scatter plot and chose the appropriate curve type. For each input sample $x_{i}$ (fertilizer application rate) and the corresponding output $t_{i}$ , we calculated the sum of the squares of the errors between wheat and maize yield and $t_{i}$ as the loss function (3), and finally selected the function with the smallest square errors as the best fitting curve.

(3) $$E\left(\omega \right)=\sum_{i=1}^{n}{\left\{{y\left(x_{i},\omega \right)-t}_{i}\right\}}^{2}$$

Here, *y* is wheat and maize yield, ω is the coefficient of $x_{i}$ , n is the number of years in the study period (*n* = 20 in this work), and *i* represents a value from 1 to *n*.

The fitting curve expressions of fertilizer use and wheat and maize yield during 2000–2020 are as follows: $Y=160,204.87-988.13\times x+2.08\times x^{2}-1.43\times 10^{-3}\times x^{3}$ (R^2^ = 0.82) and $Y=64,797.46-403.60\times x+0.91\times x^{2}-6.63\times 10^{-4}\times x^{3}$ (R^2^ = 0.89), respectively. The fitted curves had an explanatory power of 82% and 89% for the trends on the amount of chemical fertilizer and the yield of wheat and maize, respectively, and the fitting effect was good and could fully reflect the quantitative trends on the amount of chemical fertilizer and the yield.

## 3. Results

### 3.1. Crop Planting Structure

During the period from 2000 to 2020, the average annual planted area of crops in Ningjin County was 93.04 × 10^3^ ha, with the food crops and cash crops being 68.24 × 10^3^ and 24.8 × 10^3^ ha, respectively, accounting for 73.62% and 26.38% of the total planted area. Overall, the total planted area of crops fluctuated and decreased at a rate of 0.27% per year during the study period, while the planted area of food crops increased at a rate of 3.97% per year and the planted area of cash crops decreased at a rate of 8.70% per year (Figure 3). Food crops mainly included six types of crops such as wheat, maize, millets, sorghum, beans, and potatoes, with the average annual proportion of planted area (the ratio of the planted area to the total planted area of crops) being 37.12%, 34.42%, 0.09%, 0.05%, 0.44%, and 0.35%. During the study period, the planted area of wheat and maize showed a significant increase, with an increase of 18 × 10^3^ and 27.6 × 10^3^ ha, respectively. The planted area of other food crops showed a downward trend, with a decrease of 540, 292, 803, and 806 ha, respectively (Figure 3). Cash crops mainly included four types of crops such as oilseeds, cotton, vegetables, and melons, with the annual average proportion of planted area (the ratio of the planted area to the total planted area of crops) being 1.73%, 11.02%, 12.60%, and 2.12%, respectively. During the study period, the planted area of these cash crops showed a significant decreasing trend, with a decrease of 7.23 × 10^3^, 10.7 × 10^3^, 24.78 × 10^3^, and 5.05 × 10^3^ ha, representing a decrease of 96.82%, 95.03%, 80.98%, and 91.20%, respectively (Figure 3).

The average annual planted area of food crops was 68.24 × 10^3^ ha, with wheat and maize being the major crops, accounting for 97.18% of the total planted area of food crops and 71.54% of the total planted area of crops. The average annual sown area of wheat and maize throughout 2000–2020 was 35.4 × 10^3^ and 32.84 × 10^3^ ha, accounting for 61.69% and 47.96% of the total sown area of crops, and increasing at a rate of 3.42% and 5.93%, respectively. In other words, wheat and maize are the main crops in Ningjin County, and their planted areas increased from 27.52% and 15.54% in 2000 to 47.82% and 44.75% in 2020, respectively. Therefore, crop production is the main economic activity in Ningjin County, with grains being the main crop planted.

### 3.2. Major Crop Production and Security Analysis

**Figure 4:**
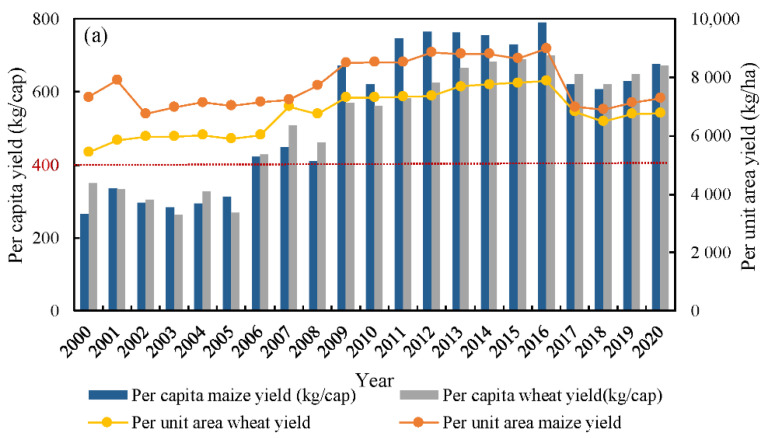

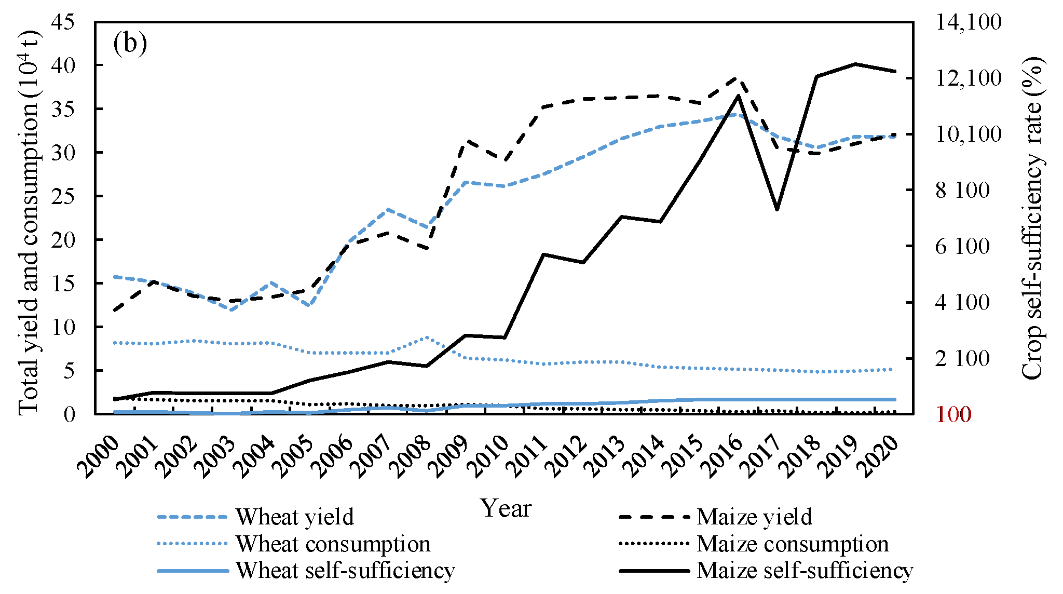
Changes in crop production and self-efficiency rate of major crops. (**a**) Depicts the per capita yield and the per unit area yield, (**b**) depicts the total yield and consumption and crop self- sufficiency rate.

Our results indicated that the average annual yield of wheat and maize in Ningjin County reached 6770.78 and 7766.79 kg/ha throughout 2000–2020, respectively. The annual yield of wheat showed a fluctuating increasing trend from 2000 to 2020 at a rate of 65.66 kg/ha, while maize showed a fluctuating decreasing trend at a rate of 2.30 kg/ha (Figure 4a). From the perspective of different years, the wheat yield presented a slightly increasing trend from 2000–2006 at a rate of 1.73%, then showed a rapid increase with a rate of 2.84% in 2007–2016, and after 2016 decreased at a rate of 3.39%. From 2000 to 2006, maize yield decreased at a rate of 0.12%, and an increase in maize yield was most notable between 2006 and 2016, with a rate of 2.35%. After 2016, the yield of maize declined rapidly at a rate of 4.51%. The trajectory of the wheat yield over the past 20 years represents the pattern of a slight increase, then a rapid increase, followed by a slow decrease. While the maize yield has generally shown a pattern of a fluctuating decrease, then a rapid increase, followed by a rapid decrease.

From the perspective of crop per capita, the annual per capita yield of wheat and maize are 519.97 and 545.42 kg/cap during 2000–2020, respectively, and both increased at rates of 16.17 and 20.56 kg/cap, respectively (Figure 4a). Compared with the FAO standard for per capita food security (400 kg/cap) [42], the per capita yield of wheat and corn in Ningjin County is relatively high, with a total of 1065.4 kg/cap, far exceeding the FAO standard, which indicates that the per capita grain yield is in a safe state. During the period from 2000 to 2005, the per capita yield of wheat and maize was low, at 308.39 and 298.15 kg/cap, respectively. From 2006 to 2020, the per capita yield significantly increased to 604.61 and 644.33 kg/cap, respectively, with the highest per capita yield of wheat and maize in 2016 reaching 700.58 and 789.40 kg/cap.

The average total yields of wheat and maize were 24.08 × 10^4^ and 25.89 × 10^4^ t throughout 2000–2020 in Ningjin County, respectively (Figure 4b). The change tendency of wheat and maize increased by rates of 5.04% and 9.03% throughout 2000–2016, respectively, and then declined by 1.21% and 4.07%, throughout 2017–2020. The average consumption of wheat and maize was 6.56 × 10^4^ and 0.91 × 10^4^ t, respectively. Wheat and maize consumption had higher values, at 7.91 × 10^4^ and 1.42 × 10^4^ t, from 2000–2008, and tended to decrease after 2008 with an average consumption at 5.55 × 10^4^ and 0.52 × 10^4^ t. As local major crops, the self-sufficiency rates of wheat and maize reached 411.25% and 5097.30%, respectively, throughout 2000–2020 (Figure 4b), which indicates that they not only can meet food self-sufficiency, but also can export for other regions with commodity rates at 73.40% and 96.50%. The self-sufficiency rate of maize showed a significant upward trend and reached 12604% at the peak in 2019. The self-sufficiency rate of wheat also showed an increasing trend, from 192.87% to 617.37% during the past 20 years.

### 3.3. Analysis of the Influencing Factors for Crop Production

Factors influencing crop production mainly include natural conditions (such as climate and soil) [13], planting structure [15], crop varieties [16], policies [45], and agricultural technology level (such as fertilizer and irrigation) [18,19]. We found that the planting structure had a significant impact on crop yield, with wheat and maize yields showing a significantly decreased trend throughout 2000–2004, and then increasing throughout 2005–2020, which was consistent with the changing trend of planting area that the wheat and maize sown areas declined by 12.96% and 0.54% between 2000 and 2004, while they grew by 7.51% and 6.42% from 2005 to 2020. This might be the case because, between 2000 and 2004, the income from grain production was significantly lower than that from other crops such as cotton and rapeseed [52]. A severe drought also occurred from 2001–2002 (Figure 2), with only 303.8 mm of annual rainfall on average, which was much less than the 452.4 mm needed for wheat [53]. Meanwhile, during the autumn planting period of 2003 (from September to October), the rainfall was as high as 288.2 mm, accounting for 40.79% of the total annual rainfall. Therefore, serious waterlogging, water accumulation, and weather conditions seriously restricted crop production, resulting in a large decrease in the sowing area.

From the perspective of farmers’ practices, the input of fertilizers and irrigation can affect grain output [21]. Intensive farming methods in Ningjin County are mainly dominated by small farmers, so the amount of fertilizer application and irrigation used by farmers during wheat and maize production processes will greatly affect grain yields [54]. Our surveys found that farmers in Ningjin County mainly used six kinds of fertilizers in wheat production in 2021, including compound fertilizer, diammonium phosphate, urea, controlled release fertilization, potassium chloride, and organic fertilizer. The gap between the actual and recommended amount of N applied for wheat in 2021 is the highest (131 kg/ha) because of its high nutrient concentration, cheap price, and reliable quality, changed by −51 and +11 kg/ha compared with 2011 and 2001, respectively (Table 2), followed by P_2_O_5_ and K_2_O, which decreased by 113 and 21 kg/ha compared with 2011 and 2001, respectively. Although the application and gap of N, P_2_O_5_, and K_2_O for wheat in 2021 decreased compared with 2011 and 2001, their use still exceeded the standard, among which P_2_O_5_ and N were always in a state of overapplication. The gap between the actual and recommended amount of N applied for maize in 2021 steadily decreased compared with 2001 and 2011, meaning that the application of N tended to be close to the standards. The amount of P_2_O_5_ applied in 2021 and 2011 exceeded the recommended amount by 89 and 91 kg/ha, respectively, but it was obviously insufficient in 2001. Therefore, P_2_O_5_ was overused for the production of maize, and the use of N and K_2_O essentially met the standard.

The main rainfall in Ningjin County is concentrated in July and August, with effective rainfall during the growth period of wheat and maize being 191.46 and 275.26 mm, respectively (Figure 2), and their water requirements are 403.73 and 358.33 mm, respectively. This means that the amount of rainfall is far lower than the required water for crops, so irrigation is an important practice to ensure wheat and maize yield. The results show that the gap between the actual and recommended amount per irrigation event for wheat in 2021 is steadily decreasing compared with 2001 and 2011 (Table 3). The total amounts of annual irrigation for wheat in 2021, 2011, and 2001 were 2907.15, 2383.99, and 2550 m^3^/ha, respectively, due to the gap of irrigation frequency resulting in that of 2021 being reasonable and those of 2011 and 2001 being insufficient. The quantities of water used by farmers for maize per irrigation event in 2021, 2011, and 2001 were 870.49, 827.14, and 945 m^3^/ha, respectively, which are all lower than that used for wheat. The gap between the actual and recommended amount per irrigation event for maize in 2021 was 195.49 m^3^/ha, which is lower than in 2001 by 74.51 m^3^/ha and higher than in 2011 by 43.35 m^3^/ha. The total amounts of annual irrigation for maize in 2021, 2011, and 2001 were 1740.98, 1248.98, and 1425 m^3^/ha, respectively, a similar gap to wheat. Generally, the irrigation frequency adopted by farmers was less than the recommendation during the cultivation season for wheat and maize, but the quantity of water used for each irrigation event was higher than the recommended amount. Therefore, a reduced irrigation frequency led to an insufficient irrigation water supply for crop growth.

**Figure 5:**
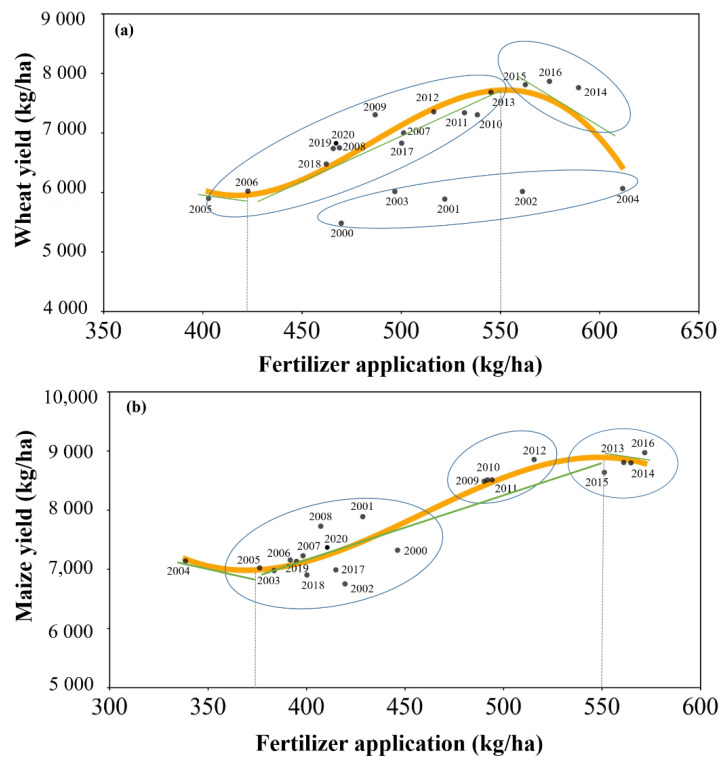
Impact of fertilizer application on crop production. (**a**) Represents the relationship between fertilizer applicaton and wheat yield, (**b**) represents the relationship between fertilizer applicaton and maize yield.

To further explore the influencing factors of crop production, we fitted the relationship between fertilizer usage and wheat and maize yields. The results showed that the impact of fertilizer application on wheat production in Ningjin County from 2000 to 2020 was close to an inverted “U” shape, and the impact of fertilizer application on maize production was close to an “S” shape. Our fitting results show that 2004 and 2016 were important turning points. The characteristics of the different time points and their influencing factors are detailed below:

Between 2000 and 2004, there was a significant negative correlation between fertilizer use and wheat yield (Figure 5). Wheat yield increased only by 10.74%, while fertilizer increased by 30.22%. Traditional planting practices made farmers believe that the more fertilizer they used, the greater the yield they would harvest [47,48]. Moreover, before 2004, farmers had to pay an agricultural tax in the form of grain; therefore, the farmers applied as much fertilizer as they could afford. The growth in fertilizer application greatly outpaced the increase in wheat yield during the same period because of the degree of agricultural knowledge and technology at the time and the low utilization efficiency of fertilizer, resulting in the formulation of a production mode characterized as high input–low output.

From 2005 to 2016, the rise in yield started to outpace the increase in fertilizer. This was primarily because the central government made it clear that it wanted to abolish the agricultural tax after 2004, and thus Ningjin County’s agricultural tax rate was reduced by 3% until the tax was abolished in 2005 [55], which has greatly reduced the production pressure of farmers, so they no longer increase yield simply by increasing fertilizer use. As a result, fertilizer use was maintained at 506 kg/ha, and high yields of wheat and maize (7174 and 8226 kg/ha) were maintained. At the same time, grain varieties have been enhanced, and local high-yielding and stable varieties such as “*Liangxing 99*”, “*Liangxing 66*” and “*Liangxing 77*” have been cultivated. With the continuous improvement in agricultural technology, the annual agricultural irrigation water consumption (174.39 million m^3^) increased by 2.1 million m^3^ compared with 2004, which improved the utilization efficiency of chemical fertilizers [34].

After 2016, the rate of chemical fertilizer application drastically reduced, and the yield trended rapidly downward. This was mostly due to the “Action Plan for Zero Growth in Fertilizer Use by 2020” outlined in Central Document No. 1 [56]. The target of zero growth of fertilizers and pesticides has been implemented nationwide. Under the unified control of national policies, the application rate of fertilizers has been effectively controlled. From 2016 to 2019, it decreased by 23.43%, while production also decreased by 16.71%. The annual mean water consumption for agricultural irrigation reduced by 29.18% over this time period compared with 2005–2016, although the trends in irrigation water quantity and grain yield were not significant (R^2^ = 0.55, sig = 0.45). Grain varieties, the prevalence of pests and diseases, and other elements impacting grain yield did not change considerably alongside the rapid decline in fertilizer use. It is evident that grain yield in Ningjin County is still strongly dependent on fertilizer.

## 4. Discussion

The NCP can produce 75% and 35% of China’s total wheat and maize output, respectively [11], and the self-sufficiency rates of wheat and maize in Ningjin County have reached 411.25% and 5097.30%, respectively, during 2000–2020, which is crucial to local and regional food security. Factors influencing crop production and security mainly include natural conditions (such as water, temperature, sunshine, and soil) [10,11], planting structure [15], crop varieties [16], agricultural policies issued by national and local governments [17] and farming practices (such as fertilizer and irrigation) [18,19]. Facing food security, especially under the background COVID-19 pandemic, China has enacted a number of policies, including a basic farmland protection policy, which has sped up the development of essential agricultural technology, and has stoked farmers’ enthusiasm for grain farming through the creation of family farms and farmers’ cooperatives. To ensure high yields and food security, the NCP has experienced significant agricultural expansion over the past few decades as a result of the long-term application of N fertilizer [10]. We also discovered that, as a grain base in Ningjin County, the sown area, production, and per capita yield of wheat and maize have significantly increased, and the yield of wheat and maize have reached production potentials of 86.07% and 98.44%, which all indicates the crop and food security stated are in a safe condition [57].

However, between 2017 and 2020, the yields of wheat and maize fell by 1.21% and 4.07%, respectively. This is mostly due to the rate of chemical fertilizer application drastically reduced after 2016 due to the “Action Plan for Zero Growth in Fertilizer Use by 2020” outlined in Central Document No. 1 [56], implying the study area’s crop yield and security greater reliance on fertilizers. Although the target of zero growth of fertilizers has been implemented nationwide and the application rate of fertilizers has been effectively controlled under the unified control of national policies, it is essentially vital to explore sustainable strategies to maintain yield while reducing environmental impacts. Therefore, scientific input ratios must be applied during crop production. By modifying the fertilization process, optimizing the fertilization structure, and enhancing the effectiveness of conventional fertilizer, the total amount of fertilizer input can be stabilized while fertilizer efficiency is increased, which can prevent the ecological environmental issues brought on by excessive use of chemical fertilizers. Achievement of high yields on the existing croplands of under-yielding nations is of great importance if global crop demand is to be met with minimal environmental impacts [21]. For example, a drip irrigation system effectively increased yield and water use efficiency, producing increases in maize of 23% and 13%, and 31% and 14% in wheat [58].

The research region has faced significant environmental concerns as a result of the last 21 years’ pursuit of high input–high yield grain production, which will eventually degrade the quality and effectiveness of grains [27,28,29]. According to our research, the yield of wheat could be improved by 26.30% when fertilizer use increases by 17.55%, and the yield of maize could be increased by 26.95% when fertilizer use increases by 34.40%. As a result, fertilizer significantly improved yield, which is in line with the findings of earlier studies [21,36,41,47,59]. However, there was no linear positive correlation between fertilizer and yield, meaning that even if fertilizer is applied to a given degree, the yield will not necessarily rise [40,47]. For instance, a recent study in China found increasing yields of maize (84%) and wheat (47%) within the range of N thresholds (150–200 and 140–210 kg ha^−1^, respectively) [44]. Our results also found that the yields of wheat and maize did not improve but instead showed a decreasing trend in Ningjin County when fertilizer exceeded 550 kg ha^−1^, which is consistent with the research results of Zhen et al. [40], Wang et al. [10], and Yang et al. [34].

Furthermore, we discovered that from 2000 to 2004, the yield and planting area of wheat both continued to decrease at rates of 12.96% and 17.01%, respectively. The main causes were waterlogging brought on by high rainfall during the planting season and dryness brought on by low rainfall during the growing season. Therefore, crop yield was impacted by the climate, which is similar to the findings of Wang et al. [60] and Liu et al. [58]. Climate change will not only affect crop yield, but also farmers’ practices. for example, drought will affect farmers’ irrigation frequency and amount. Wang et al. [60] indicated that farmers will adjust their irrigation practices when faced with a severe drought by enhancing irrigation intensity and efficiency to lessen the consequences of such drought [60]. We found that the irrigation frequency adopted by farmers was less than the recommended during the cultivation season with an uneven distribution of rainfall, which may lead to insufficient irrigation water supply for crop growth. However, due to the higher amount per irrigation event resulting in the total irrigation quantity being higher than the recommended amount, therefore, irrigation should be carried out in accordance with the water demand of crops in different periods [61], and the amount of irrigation each time should be based on climatic conditions (e.g., rainfall, evaporation) [62,63], reasonable irrigation should be conducted by government departments, especially agricultural guidance centers [64,65].

Irrigation and fertilization application not only contribute to crop yield but also result in a series of environmental issues [27,28,29]. According to Yang et al. [31], the North China Plain’s agricultural productivity is seriously threatened by severe water scarcity, particularly during the winter wheat growing season (October–June) [66]. The reason is that the precipitation can only provide about 25–40% of the water needed for the entire wheat-growing season [67]; therefore, more than 70% of irrigation water is extracted from groundwater through pump wells during the wheat growing season, which results in groundwater net consumption surpassing 300 mm y^−1^ [68] and a dramatic decrease in the underground water table with 0.88 mm y^−1^ [23]. In addition, excessive use of fertilizers, particularly nitrogen fertilizer, has also had a negative impact on groundwater quality and nitrate contamination. Wang et al. [10] revealed that the agricultural N emission to water bodies was 1079 Gg in 2012 accounted for 54% of total N emission. Zhen et al. [40] showed that nitrate levels ranged from 55 to 180 mg L^−1^ in Ningjin County, higher than the recommended level for drinking water (50 mg L^−1^). Min et al. [68] also found that nitrogen leaching losses have been estimated to be 22.4 and 5.2 kg/ha for winter wheat and summer maize, respectively [57]. Therefore, it is necessary to guide farmers scientifically and convert their farming concepts and practices from traditional to environmentally friendly, such as enhancing the application of crop residues and manure instead of chemical fertilizer [69], introducing legumes [70], and organic farming [71]. It would be a practical approach to involve Ningjin County in national high-standard farmland construction and planning, and to apply scientific planting and management principles, considering how to increase productivity, improve quality, and protect the environment where agricultural production takes place.

## 5. Conclusions

We discovered that throughout 2000–2021, the production of wheat and maize in Ningjin County increased and the crop security stated in a safe condition, with this trend being driven by planting area, policies, crop varieties, climate, and farmer practices, particularly rainfall, irrigation, and fertilization. Specifically, this included the following conclusions:

(1) The average sown area of wheat and maize during 2000–2020 accounted for 61.69% and 47.96% of the total sown area of crops, and increased at a rate of 3.42% and 5.93%, respectively. Therefore, wheat and maize are the main crops in Ningjin County, and their planted areas increased from 27.52% and 15.54% in 2000 to 47.82% and 44.75% in 2020, respectively.
(2) The annual per capita yield of wheat and maize are 519.97 and 545.42 kg/cap throughout 2000–2020 respectively, indicating that the per capita grain yield is in a safe state. The self-sufficiency rate of maize showed a significant upward trend and reached 12604% at the peak in 2019, the self-sufficiency rate of wheat also showed an increasing trend, from 192.87% to 617.37%, which also indicates that wheat and maize can meet food self-sufficiency.
(3) Throughout 2000–2020, trends in fertilizer and wheat yield approximately showed an inverted “U” shape, and that of fertilizer and maize yield had an “S” shape, meaning that as fertilizer use increased, grain yield first rapidly increased before becoming essentially stable or decreasing. The results showed that 550 kg/ha is the threshold of fertilizer application, and exceeding this level will enter the stage of diminishing marginal returns. The national agricultural production and environmental protection policies, continuous improvement of crop varieties, as well as the farmers’ traditional practices have significant impacts on crop production.

## Figures and Tables

**Table 1:** Contents of N, P_2_O_5_, and K_2_O in different fertilizers.

Name of Chemical Fertilizer	Contents * (%)		
	N	P_2_O_5_	K_2_O
*Three element compound fertilizer*	15	5	20
*Diammonium phosphate*	15	47	-
*Ammonium bicarbonate*	17.1	-	-
*Urea*	46	-	-
*Potassium chloride*	-	-	63
*Superphosphate*	-	17	-
*Controlled release fertilizer*	15	9	12
*Three element compound fertilizer*	15	5	20

* The conversion coefficient was determined using the fertilizer name discovered through field research, together with the atomic weight and molecular formulae of N, P_2_O_5_, and K_2_O.

**Table 2:** The gap between actual and recommended amount of fertilizer application by crops (kg/ha).

Crops	Fertilizer Application	2021			2011			2001		
		N	P_2_O_5_	K_2_O	N	P_2_O_5_	K_2_O	N	P_2_O_5_	K_2_O
Wheat	A.D.	311 *	181	75 *	392	284	81	375	150	45
	R.D.	150–180	60–100	40–60	180–210	75–90	75–90	210–255	90–120	7–9
	Gap	+131	+81	+15	+182	+194	&	+120	+30	+36
Maize	A.D.	182 *	164 *	70 *	248	181	47	240	45	15
	R.D.	150–180	60–75	60–70	200–225	80-90	150–180	165–210	90–105	5–7
	Gap	+2	+89	&	+23	+91	−103	+30	−45	+8

Note: A.D. = actual dosage applied by farmers, R.D. = recommended dosage. Gap means that compared with the high values of R.D., + represents exceeding R.D., − represents insufficient compared to R.D., & represents within the scope of R.D. * One-sample *t*-test significant at the 0.05 level.

**Table 3:** Irrigation water applied by farmers versus recommended rates.

Items		2021		2011		2001	
		Wheat	Maize	Wheat	Maize	Wheat	Maize
Amount of per irrigation event (m^3^/ha)	Act.	969.05	870.49	1083.63	827.14	975	945
	Rec.	750–900	525–675	716–850	525–675	600–750 [1]	525–675
	Gap	+219.05	+195.49	+333.63	+152.14	+225	+270
Total amount of annual irrigation (m^3^/ha)	Act.	2907.15	1740.98	2383.99	1248.98	2550.00	1425.00
	Rec.	2400–3200 [3]	1350–2100	2864–3400 [2]	1425–2300	2850–3000	1500–2000
	Gap	&	&	−480.01	−176.02	−300.00	−75.00
Irrigation frequency(times/y)	Act.	3	2	2	2	3	2
	Rec.	2–3	2–4	4–5	3–4	4–6	3–5
	Gap	&	&	-	-	-	-

Note: Act. = actual amount applied by farmers, Rec. = recommended rate. Gap means that compared with the high values of Rec., + represents exceeding Rec., − represents insufficient compared to Rec., & represents within the scope of Rec.

## Data Availability

Data is contained within the article or supplementary material.

## Supplementary Materials

The following supporting information can be downloaded at: https://www.mdpi.com/2304-8158/12/11/2196/s1, Supplemental questionnaire: Questionnaire of farmers in Ningjin County, Dezhou City, Shandong Province, 2021.

## References

[1] United Nations (UN) (2015). Transforming Our World: The 2030 Agenda for Sustainable Development.

[2] Cui Z.L., Zhang H.Y., Chen X.P., Zhang C.C., Ma W.Q., Huang C.D., Zhang W.F., Mi G.H., Miao Y.X., Li X.L. (2018). Pursuing sustainable productivity with millions of smallholder farmers. Nature.

[3] Food and Agriculture Organization (FAO) (2021). The State of Food and Agriculture 2021: Making Agri-Food Systems More Resilient to Shocks and Stresses. https://www.fao.org/3/CB4476EN/online/CB4476EN.htm.

[4] Laborde D., Martin W., Swinnen J., Vos R. (2020). COVID-19 risks to global food security. Science.

[5] Knorr D., Khoo C.S.H. (2020). COVID-19 and Food: Challenges and Research Needs. Front. Nutr..

[6] Niu Y.N., Xie G.D., Xiao Y., Liu J.Y., Zou H.X., Qin K.Y., Wang Y.Y., Huang M.D. (2022). The story of grain self-sufficiency: China’s food security and food for thought. Food Energy Secur..

[7] Food and Agriculture Organization (FAO) (2021). Crop Prospects and Food Situation—Quarterly Global Report No. 1, March 2021.

[8] National Bureau of Statstics (2020). Statistical Communique of the People’s Republic of China on the 2020 National Economic and Social Development. http://www.stats.gov.cn/tjsj/zxfb/202202/t20220227_1827960.html.

[9] Kang S., Eltahir E.A.B. (2018). North China Plain threatened by deadly heatwaves due to climate change and irrigation. Nat. Commun..

[10] Wang S.Q., Hu Y.K., Yuan R.Q., Feng W.Z., Pan Y., Yang Y.H. (2019). Ensuring water security, food security, and clean water in the North China Plain—Conflicting strategies. Curr. Opin. Environ. Sustain..

[11] Meng Q.F., Sun Q.P., Chen X.P., Cui Z.L., Yue S.C., Zhang F.S., Römheld V. (2012). Alternative cropping systems for sustainable water and nitrogen use in the North China Plain. Agric. Ecosyst. Environ..

[12] Lu W.C. (2002). Effects of agricultural market policy on crop production in China. Food Policy.

[13] Zurayk R. (2020). Pandemic and food security: A view from the global south. J. Agric. Food Syst. Commun. Dev..

[14] Bali N., Singla A. (2022). Emerging Trends in Machine Learning to Predict Crop Yield and Study Its Influential Factors: A Survey. Arch. Comput. Methods Eng..

[15] Wei X., Zhang Z., Wang P., Tao F. (2017). Recent patterns of production for the main cereal grains: Implications for food security in China. Reg. Environ. Chang..

[16] Zhang Q., Wang K.Y., Han Y.Y., Liu Z.Q., Yang F., Wang S.F., Zhao X.Y., Zhao C.J. (2022). A crop variety yield prediction system based on variety yield data compensation. Comput. Electron. Agric..

[17] Wuepper D., Le Clech S., Zilberman D., Mueller N., Finger R. (2020). Countries influence the trade-off between crop yields and nitrogen pollution. Nat. Food.

[18] Wang M., Ma L., Strokal M., Chu Y., Kroeze C. (2018). Exploring nutrient management options to increase nitrogen and phosphorus use efficiencies in food production of China. Agric. Syst..

[19] Li M., Singh V.P. (2019). Sustainability of water and energy use for food production based on optimal allocation of agricultural irrigation water. Int. J. Water Resour. Dev..

[20] Yu C.Q., Huang X., Chen H., Godfray H.C.J., Wright J.S., Hall J.W., Gong P., Ni S.Q., Qiao S.C., Huang G.R. (2019). Managing nitrogen to restore water quality in China. Nature.

[21] Tilman D., Balzer C., Hill J., Befort B.L. (2011). Global food demand and the sustainable intensification of agriculture. Proc. Natl. Acad. Sci. USA.

[22] Mueller N.D., Gerber J.S., Johnston M., Ray D.K., Ramankutty N., Foley J.A. (2012). Closing yield gaps through nutrient and water management. Nature.

[23] Du T.S., Kang S.Z., Zhang X.Y., Zhang J.H. (2014). China’s food security is threatened by the unsustainable use of water resources in North and Northwest China. Food Energy Secur..

[24] Ju X., Xing G., Chen X., Zhang S.L., Zhang L.J., Liu X.J., Cui Z.L., Yin B., Christie P., Zhu Z.L. (2009). Reducing environmental risk by improving N management in intensive Chinese agricultural systems. Proc. Natl. Acad. Sci. USA.

[25] Wang Q.L., Bai Y.H., Gao H.W., He J., Chen H., Chesney R.C., Kuhn N.J., Li H.W. (2008). Soil chemical properties and microbial biomass after 16 years of no-tillage farming on the Loess Plateau. Geoderma.

[26] Chen X., Cui Z., Vitousek P.M., Cassman K.G., Matson P.A., Bai J.S., Meng Q.F., Hou P., Yue S.C., Romheld V. (2011). Integrated soil-crop system management for food security. Proc. Natl. Acad. Sci. USA.

[27] Wang M.R., Ma L., Strokal M., Ma W.Q., Liu X.J., Kroeze C. (2018). Hotspots for Nitrogen and Phosphorus Losses from Food Production in China: A County-Scale Analysis. Environ. Sci. Technol..

[28] Chen X.P., Cui Z.L., Fan M.S., Vitousek P., Zhao M., Ma W.Q., Wang Z.L., Zhang W.J., Yan X.Y., Yang J.C. (2014). Producing more grain with lower environmental costs. Nature.

[29] Guo J.H., Liu X.J., Zhang Y., Shen J.L., Han W.X., Zhang W.F., Christie P., Goulding K.W.T., Vitousek P.M., Zhang F.S. (2010). Significant Acidification in Major Chinese Croplands. Science.

[30] Wei X.Q., Ye Y., Zhang Q., Li B.B., Wei Z.D. (2019). Reconstruction of cropland change in North China Plain Area over the past 300 years. Glob. Planet Chang..

[31] Yang G.Y., Li S.Y., Wang H., Wang L. (2022). Study on agricultural cultivation development layout based on the matching characteristic of water and land resources in North China Plain. Agric. Water Manag..

[32] Sun Q.P., Krobel R., Muller T., Romheld V., Cui Z.L., Zhang F.S., Chen X.P. (2011). Optimization of yield and water-use of different cropping systems for sustainable groundwater use in North China Plain. Agric. Water Manag..

[33] Wang G.L., Chen X.P., Cui Z.L., Yue S.C., Zhang F.S. (2014). Estimated reactive nitrogen losses for intensive maize production in China. Agric. Ecosyst. Environ..

[34] Yang Z.H., Hu Y., Zhang S., Raza S., Wei X.R., Zhao X.N. (2022). The Thresholds and Management of Irrigation and Fertilization Earning Yields and Water Use Efficiency in Maize, Wheat, and Rice in China: A Meta-Analysis (1990–2020). Agronomy.

[35] Hartmann T.E., Yue S.C., Schulz R., He X.K., Chen X.P., Zhang F.S. (2015). Yield and N use efficiency of a maize–wheat cropping system as affected by different fertilizer management strategies in a farmer’s field of the North China Plain. Field Crop. Res..

[36] Su X., Ping J., Ye Q. (2011). Terrestrial water variations in the North China Plain revealed by the GRACE mission. Sci. China Earth.

[37] Feng W., Zhong M., Lemoine J.M., Biancale R., Hsu H.T., Xia J. (2013). Evaluation of groundwater depletion in North China using the Gravity Recovery and Climate Experiment (GRACE) data and ground-based measurements. Water Resour. Res..

[38] Wang H.Y., Zhang Y.T., Chen A.Q., Liu H.B., Zhai L.M., Lei B.K., Ren T.Z. (2017). An optimal regional nitrogen application threshold for wheat in the North China Plain considering yield and environmental effects. Field Crop. Res..

[39] Liu F., Zhen P.N., Wang S. (2022). Groundwater quality assessment and health risks from nitrate contamination in the Heilongdong Spring Basin, a typical headwater basin of the North China Plain. Environ. Sci. Pollut. Res..

[40] Zhen L., Zoebisch M.A., Chen G.B., Feng Z.M. (2006). Sustainability of farmers’ soil fertility management practices: A case study in the North China Plain. J. Environ. Manag..

[41] Rana R.L., Bux C., Lombardi M. (2022). Trends in scientific literature on the environmental sustainability of the artichoke (*Cynara cardunculus* L. spp.) supply chain. Br. Food J..

[42] Vågsholm I., Arzoomand N.S., Boqvist S. (2020). Food Security, Safety, and Sustainability-Getting the Trade-Offs Right. Front. Sustain. Food Syst..

[43] Denning G., Kabambe P., Sanchez P., Malik A., Flor R., Harawa R., Nkhoma P., Zamba C., Banda C., Magombo C. (2009). Input Subsidies to Improve Smallholder Maize Productivity in Malawi: Toward an African Green Revolution. PLoS Biol..

[44] Barrett C.B. (2010). Measuring food insecurity. Science.

[45] Ningjin County Bureau of Statistics, 2000–2020. http://www.sdningjin.gov.cn/n55034037/n55034392/n55034412/n56369810/index.html.

[46] (2020). National Development and Reform Commission(NDRC). https://navi.cnki.net/knavi/yearbooks/index?uniplatform=NZKPT.

[47] Zhen L., Routray J.K., Zoebisch M.A., Chen G.B., Xie G.D., Cheng S.K. (2005). Three dimensions of sustainability of farming practices in the North China Plain—A case study from Ningjin County of Shandong Province, PR China. Agric. Ecosyst. Environ..

[48] Zhen L., Routray J.K. (2002). Groundwater resource use practices and implications for sustainable agricultural development in the North China Plain: A case study in Ningjin County of Shandong Province, PR China. Int. J. Water Resour. Dev..

[49] Shandong Province Bureau of Statistics, 2000–2020. http://tjj.shandong.gov.cn/col/col6279/index.html.

[50] Pei S.C., Horng J.H. (1995). Fitting digital curve using circular arcs. Pattern Recognit..

[51] El-Mikkawy M., Atlan F. (2013). Remarks on two symmetric polynomials and some matrices. Appl. Math Comput..

[52] Peng L.J., Lu G., Pang K., Yao Q. (2021). Optimal farmer’s income from farm products sales on live streaming with random rewards: Case from China’s rural revitalisation strategy. Comput. Electron. Agric..

[53] Yang X.L., Chen Y.Q., Pacenka S., Gao W.S., Ma L., Wang G.Y., Yan P., Sui P., Steenhuis T.S. (2015). Effect of diversified crop rotations on groundwater levels and crop water productivity in the North China Plain. J. Hydrol..

[54] Andersson Djurfeldt A. (2015). Urbanization and linkages to smallholder farming in sub-Saharan Africa: Implications for food security. Glob. Food Secur..

[55] The State Council of the People’s Republic of China (2004). The State Council on Accomplishing the Pilot Work of Deepening the Taxes and Fees Reform in Rural Areas in 2004. http://www.gov.cn/zhengce/content/2008-03/28/content_1982.htm.

[56] The General Office of the Communist Party of China Central Committee and the General Office of the State Council (2016). Zero Growth in the Amount of Chemical Fertilizers and Pesticides Used in Major Crops by 2020. http://www.moa.gov.cn/govpublic/ZZYGLS/201505/t20150525_4614695.htm.

[57] Zhang B.B., Li X., Chen H.B., Niu W.H., Kong X.B., Yu Q., Zhao M.J., Xia X.L. (2022). Identifying opportunities to close yield gaps in China by use of certificated cultivars to estimate potential productivity. Land Use Policy.

[58] Liu Y.J., Chen Q.M., Tan Q.H. (2019). Responses of wheat yields and water use efficiency to climate change and nitrogen fertilization in the North China plain. Food Secur..

[59] FAO FAOSTAT-Agriculture Database. http://faostat.fao.org/site/339/default.aspx.

[60] Wang J.X., Yang Y., Huang J.K., Adhikari B. (2019). Adaptive irrigation measures in response to extreme weather events: Empirical evidence from the North China plain. Reg. Environ. Chang..

[61] Fawzy S., Osman A.I., Doran J., Rooney D.W. (2020). Strategies for mitigation of climate change: A review. Environ. Chem. Lett..

[62] Yang X.L., Song Z.W., Wang H., Shi Q.H., Chen F., Chu Q.Q. (2012). Spatio-temporal variations of winter wheat water requirement and climatic causes in Huang-Huai-Hai Farming Region. Chin. J. Eco-Agric..

[63] MO Y.C., Bao Z.X., Song X.M., Wang G.Q., Liu C.S., Tian Y.M. (2022). Study on the Spatio-temporal Evolution of Water Demand and Water Shortage of Main Crops in Huang-Huai-Hai Basin from 1961 to 2017. J. N. China Univ. Water Resour. Electr. Power.

[64] Ncube B. (2018). Constraints to smallholder agricultural production in the Western Cape, South Africa. Phys. Chem. Earth.

[65] Phiri A., Chipeta G.T., Chawinga W.D. (2019). Information needs and barriers of rural smallholder farmers in developing countries A case study of rural smallholder farmers in Malawi. Inform. Dev..

[66] Liu Y.B., Lim T.C., Wang Y. (2001). Vibration characteristics of welded beam and plate structures. Noise Control. Eng. J..

[67] Iqbal M.A., Shen Y., Stricevic R., Pei H., Sun H., Amiri E., Penas A., del Rio S. (2014). Evaluation of the FAO Aqua Crop model for winter wheat on the North China Plain under deficit irrigation from field experiment to regional yield simulation. Agric. Water Manag..

[68] Min L., Shen Y., Pei H., Wang P. (2018). Water movement and solute transport in deep vadose zone under four irrigated agricultural land-use types in the North China Plain. J. Hydrol..

[69] Zhang X., Qian H., Hua K., Chen H., Deng A., Song Z., Zhang J., Raheem A., Danso F., Wang D. (2022). Organic amendments increase crop yield while mitigating greenhouse gas emissions from the perspective of carbon fees in a soybean-wheat system. Agric. Ecosyst. Environ..

[70] Bux C., Varese E., Lombardi M., Amicarelli V. (2022). Economic and Environmental Assessment of Conventional versus Organic Durum Wheat Production in the Mediterranean Area. Sustainability.

[71] Liao P., Sun Y., Zhu X., Wang H., Wang Y., Chen J., Zhang J., Zeng Y., Zeng Y., Huang S. (2021). Identifying agronomic practices with higher yield and lower global warming potential in rice paddies: A global meta-analysis. Agric. Ecosystic. Environ..

