# Supplementary material for: Crop Production and Security in Ningjin County of the North China Plain
Source: Foods. 2023 May 30;12(11):2196. doi: 10.3390/foods12112196 (PMC10252609; doi:10.3390/foods12112196)
Supplement: Supplementary file 1 [file foods-12-02196-s001.zip › foods-2385965-supplementary.pdf]

Questionnaire of farmers in Ningjin County, Dezhou City, Shandong Province, 2021

________（Town）_______Villages_ Date：___Month____Day__Year Investigator:______Name of Respondent:_________Tel：___

1. Please fill in the basic information about the family members.

No.	Gender	Age*	Degree of education **	Profession			
				①Cultivation	②Breeding	③Wage	④Other
1				_____day/year,_____yuan/day			
2				_____day/year,_____yuan/day			
3				_____day/year,_____yuan/day			
4				_____day/year,_____yuan/day			
5				_____day/year,_____yuan/day			

*Age(years):①＜18②[18-30]③（30-40]④(40-50]⑤(50-60]⑥＞60 **Degree of education: ①primary school ②middle school ③high school ④College degree or above

2. Please fill in the following form as required

	Name of crops	Cultivated area			Per unit yield			Irrigation							Ground cover condition	
		Last year (mu)	10 years ago(mu)	Changing reason	Last year (jin/mu)	10 years ago(jin/mu)	Changing reason	① Yes ② No	Methods *	Period**	Source ***	Distance from source (m)	Annual number of irrigation(times)	Hous of each irrigation (hr/time)	Area covered by mulch(mu)	Area of plastic shelter(mu)
1	Wheat															
2	Maize															
3	Cotton															
4	Vegetables															
3	Others															

*Irrigation methods:①traditional irrigation(broad irrigation)②sprinkling irrigation③trickle irrigation④border irrigation⑤Other **Irrigation period:①sowing②emergence③tillering④seedling establishment⑤elongation stage⑥heading⑦anthesis ***Source of irrigation:①river②well③reservoir④industrial wastewater treatment. 1jin = 1/2 kilogram; 1 hectare = 1 mu.

3. Number of irrigation wells in your home: ___items
4. Is there enough irrigation water available? ( ) Yes ( ) No
5. Compared with 10 years ago, irrigation water consumption has ( ) increased ( ) decreased ( ) remained unchanged?
6. Is there any contamination of water sources (such as rivers, wells, reservoirs, etc.) in your village? ( ) None, ( ) Yes

Pollution sources	Changes of water source pollution in recent 10 years *	Will affect food production and income **
Industrial waste		
Fertilizer use		
Pesticide use		
Human and animal excreta		

*Changes of water source pollution in recent 10 years: ①increased ②decreased ③unchanged ** Will affect food production and income: ①Yes ②No

7. Is the farmland contracted by your family enough at present? () Yes () No. Are you willing to contract more farmland?
（ ）Yes, the reason is:___________________
（ ） No, the reason is:___________________
8. Do you think these farmland are yours?
（1）Yes
（2）No. Who do you think it belongs to? A. town/village committee B. country C. don't know
9. Is the contracted farmland transferred to others?
（1）Yes. Transfer area_____mu; Transfer year_____; Transfer to（）neighbor（）large scale growers （）enterprise （）cooperative
（2）No. （）grow it ownself （）leave uncultivated
10. Is the farmland currently contracted leased to others?
(1)No. （）grow it ownself （）leave uncultivated
(2)Yes. Do you sign formal contracts? Rent ____ yuan/mu/year; The lease area is _____ mu; The lease is for ____ years; Lease to（）neighbor（）large scale growers （）enterprise （）cooperative
11. Do you think there has been any change in the degree of cracking and hardening of cultivated land in recent 10 years?
（ ）aggravating （ ） unchanged （ ）mitigating
What is the change of farmland fertility?
（ ）decreasing （ ）unchanged （ ）improving
12. Could you describe the crop straw returning to the field?

Choice	Amount of straw returning to the field

	Wheat	Maize	Other
1/3			
1/2			
All			
None*			

* Use of non-returning crop straw:①for fuel②feed stocks③sell to paper mills④sell to feed mills/farmers⑤other

13. Please fill in the following table with the fertilizer usage of the major crops last year

Type and name of fertilizer	Wheat				Maize			
	Total application (jin/mu)	Frequency (times/year)	Variation of dosage *	Period **	Total application(jin/mu)	Frequency (times/year)	Variation of dosage*	Period **
**Organic fertilizer** 1.Cow dung								
2.Barnyard manure								
3.Straw								
4.Fecal								
5.Other								
**Chemical fertilizer** 1. Compound fertilizer								
2.Diammonium phosphate								
3.Ammonium bicarbonate								
4.Urea								
5. Superphosphate								
6. Potassium chloride								
7.Controlled release fertilizer								
8.Other								

* Compared with 10 years ago, the amount of fertilizer per mu changed: ①increased ②decreased ③unchanged

**fertilizer appllied period:①sowing②emergence③tillering④seedling establishment⑤elongation stage⑥heading⑦anthesis

14. Do you know how much fertilizer should be applied to different crops per mu?

（1） Yes. From where to know?

（ ）agricultural technology extension department （ ）village head （ ）neighbor （ ）fertilizer sales company

（ ）read books about （ ）instructions for packaging bag （ ）TV/ phone and other electronic devices

（2） No. How do you know how much fertilizer to use?

（ ）by experience （ ）apply as much fertilizer as you buy （ ）instructions for packaging bag

15. Do you think the amount of fertilizer is appropriate? ( ) suitable ( ) too much ( ) too little
16. How do you fertilize?

（ ）broadcast（ ）machine applied （ ）drilling（ ）hole fertilization（ ）trickle irrigation（ ）spray（ ）other

17. Do you think the more fertilizer you apply, the higher the yield? ( ) Yes ( ) No
18. Do you think too much fertilizer will pollute the farmland or groundwater? ( ) Yes ( ) No
19. Please describe the occurrence of major diseases and the use of pesticides last year

Crops	Major diseases	Pesticides *	Dosage of pesticides(jin/mu)	Frequency(times/year)	Change of dosage**	Is the dosage appropriate?***
Wheat	1.stripe rust					
	2.head scab					
	3.sharp eyespot					
	4.aphid					
	5.powdery mildew					
Maize	1.spodoptera frugiperda					
	2.armyworm					
	3.borer					
	4.leaf blight					
	5.smut					
	6.sharp eyespot					
Others						

* Name of pesticides:①pyrethroid②carbendazim③imidacloprid④acaricide⑤omethoate⑥acetaniprid⑦lesiben⑧triadimefon⑨propiconazole⑩lipoic acid

** Compared with 10 years ago, the amount of pesticides per mu changed: ①increased ②decreased ③unchanged *** Is the dosage appropriate? ①suitable ②too much ③too little

20. Do you know the standard amount of pesticides for different crop diseases?

（1）Yes.。From where to know？

（ ）agricultural technology extension department （ ）village head （ ）neighbor （ ）pesticides sales company

（ ）read books about （ ）instructions for packaging bag （ ）TV/ phone and other electronic devices

（2）No. How do you know how much pesticides to use?

（ ）by experience （ ）apply as much fertilizer as you buy （ ）instructions for packaging bag

21. Do you think the more pesticides you use, the less pests you have? ( ) Yes ( ) No
22. Do you think the use of pesticides will cause pollution to farmland or water? ( ) Yes ( ) No
23. Please describe your usual method of dosing

（ ）spray （）seed dressing （ ）mix with manure （ ）mix with irrigation water

（ ）aerosol （ ）dusting （ ）dip the seed （ ）coating （ ）other

24. Do you use protection when using pesticides? () Yes, there is ______________ () No, why?
25. Does anyone in your family have health problems due to the use of chemical fertilizers or pesticides?

（1）None.

（2）Yes, for example, _____________. Has the phenomenon increased or decreased over the past decade?

26. Please fill in the production cost of main crops in your cultivated land last year in the following table (yuan).

Items	Wheat	Maize	Others	Total
seeds				
organic fertilizers				
chemical fertilizers				
pesticides				
hire farm machinery/livestock				
transportation *				
cost of building plastic shelter/mulch				
labor input **				
others				

* transportation costs include the cost of moving products, selling products, etc., such as selling trees

** The number of people employed, the number of days employed (___ days/year), the wage (___ yuan/day) and the total expenses of the employer

27. Fill in the following table itemized and detailed your income from arable land last year

Source of income	Income	Source of income	Income(yuan)
sale of wheat	quantity sold：_______t	Non-farm income	
	income：______yuan	-food subsidies	
sale of maize	quantity sold：_______t	- other subsidies	
	income：______yuan	Others	
Total：			

28. Have you ever received the services of an agricultural extension worker? If so, fill out the form below

Services received	Times of serrvices	Services organizations(play check √)					
		Village-level	Town-level	County-level	Peasants' cooperative	Town extension	Others
1.							
2.							
3.							
